# A stacking machine learning model for identifying comorbid anxiety disorder in children and adolescents with functional abdominal pain disorders

**DOI:** 10.3389/fpsyt.2026.1904174

**Published:** 2026-10-23

**Authors:** Weiwei Wu, Quanquan Ye, Xiaochun Lin, Sisi Li, Shishi Wang, Xiaoyan Liu

**Affiliations:** Department of Pediatric Gastroenterology, The Second Affiliated Hospital & Yuying Children’s Hospital of Wenzhou Medical University, Wenzhou, Zhejiang, China

**Keywords:** anxiety disorder, children and adolescents, functional abdominal pain disorders, LASSO, machine learning, SHAP, stacking ensemble

## Abstract

**Objective:**

Comorbid anxiety disorder is common in children and adolescents with functional abdominal pain disorders, but clinically usable risk-stratification tools integrating abdominal pain, psychosocial, school-related, and family-level information remain limited.

**Methods:**

In this observational predictive modeling study, children and adolescents aged 7–18 years with Rome IV-defined functional abdominal pain disorders were evaluated between December 2021 and December 2025. Participants were randomly allocated at a 7:3 ratio into training and internal validation cohorts using stratified sampling by anxiety status. Candidate predictors included demographic, abdominal pain-related, somatic, psychosocial, school-related, and family-level variables. After LASSO-based feature selection, four prespecified base learners—explainable boosting machine, calibrated support vector machine, random forest, and CatBoost—were combined using an L2-penalized logistic regression stacking meta-learner. Performance was assessed using discrimination, threshold-dependent metrics, calibration, decision curve analysis, learner diversity, and SHAP-based interpretability.

**Results:**

Among 629 eligible patients, 210 (33.4%) had comorbid anxiety disorder. The training cohort included 440 patients, and the validation cohort included 189 patients, with similar anxiety prevalence in both cohorts. Clinically relevant correlates of anxiety included older age, longer symptom duration, higher pain frequency, night-time awakening, pain temporally associated with stressful events, higher SDSC and CSI-24 scores, higher parental GAD-7 score, and greater school absence. In out-of-fold training estimates, the stacking model achieved an AUC of 0.831 and a Brier score of 0.151. In the validation cohort, it showed the highest numerical performance among candidate models, with an AUC of 0.846 (95% CI, 0.789–0.894), accuracy of 0.788, sensitivity of 0.762, specificity of 0.802, F1 score of 0.706, and Brier score of 0.146. Decision curve analysis suggested potential clinical utility across selected threshold ranges. SHAP analysis identified school absence, parental GAD-7 score, CSI-24 score, pain-stress association, symptom duration, pain frequency, SDSC score, age, and night-time awakening as leading contributors to the final model output.

**Conclusion:**

A stacking machine learning model integrating clinical, psychosocial, school-related, and family-level variables showed acceptable internal-validation performance for identifying comorbid anxiety disorder in children and adolescents with functional abdominal pain disorders. External validation is needed before clinical implementation.

## 1 Introduction

Functional abdominal pain disorders are among the most frequent causes of chronic or recurrent abdominal pain in children and adolescents and are now regarded as disorders of gut brain interaction rather than isolated gastrointestinal complaints (1–4). Under the Rome IV framework, these disorders include functional dyspepsia, irritable bowel syndrome, abdominal migraine, and functional abdominal pain not otherwise specified (5–8). Although their diagnostic definition is symptom based, their clinical expression is often shaped by multiple interacting domains, including pain burden, sleep quality, somatic symptom amplification, school functioning, and family psychological context (9, 10). Anxiety is particularly relevant in this population. It may intensify pain perception, increase health care seeking, impair daily functioning, and contribute to persistence of symptoms, while abdominal pain itself can reinforce anticipatory worry, avoidance, and school absence (11, 12). In pediatric gastroenterology practice, the clinical presentation is therefore rarely confined to pain frequency or pain intensity. Children may present with nausea, abdominal bloating, night time awakening, recurrent school absence, academic stress, or family distress, all of which may coexist with anxiety but are not always recognized as part of the same risk profile (13, 14). The early identification of anxiety in children and adolescents with functional abdominal pain disorders is clinically important because psychological comorbidity can influence symptom severity, treatment response, and long term functional outcomes. However, anxiety may be under recognized when the presenting complaint is abdominal pain, especially in settings where mental health assessment is not routinely embedded in gastroenterology care.

Current approaches to identifying anxiety in children and adolescents with functional abdominal pain disorders remain limited (15, 16). Standardized anxiety instruments are useful for screening and diagnosis, but they do not fully address the broader clinical need to identify patients who may warrant specialist psychological assessment. Previous studies have linked anxiety with pain severity, sleep disturbance, somatic symptoms, school impairment, parental anxiety, and adverse family context; however, these factors are often assessed separately or within linear models that may not capture their combined and potentially nonlinear effects. Machine learning offers a way to integrate multidimensional clinical information and model complex predictor structures, but clinical applications remain vulnerable to overfitting, information leakage, poor calibration, limited interpretability, and insufficient assessment of clinical utility. These concerns are particularly relevant in pediatric studies, where sample sizes are often moderate and models must remain transparent enough to support clinical decision making.

To address these gaps, we developed and internally validated a stacking machine learning model to identify comorbid anxiety disorder in children and adolescents with functional abdominal pain disorders. The model integrated routinely obtainable demographic, abdominal pain-related, somatic, sleep, school, psychosocial, and family-level variables. Four prespecified base learners—explainable boosting machine, calibrated support vector machine, random forest, and CatBoost—were combined using an L2-penalized logistic regression meta-learner trained on out-of-fold predicted probabilities after LASSO-based feature selection. To reduce information leakage, all preprocessing, feature selection, tuning, calibration, and threshold selection were performed within the training pipeline, with the validation cohort reserved for final evaluation. Model performance was assessed using discrimination, calibration, Brier score, threshold-dependent metrics, decision curve analysis, learner diversity, and SHAP-based interpretability. The model was intended to support, rather than replace, clinical assessment by identifying patients who may warrant more detailed psychological evaluation.

## 2 Methods

### 2.1 Study design, setting, and participants

This observational predictive modeling study was conducted among children and adolescents with functional abdominal pain disorders who were evaluated between December 2021 and December 2025. The study was designed to develop and internally validate a machine learning stacking model for identifying comorbid anxiety disorder in this clinical population. Eligible patients were retrospectively identified from the electronic medical records of The Second Affiliated Hospital & Yuying Children’s Hospital of Wenzhou Medical University, and all clinical, psychosocial, school-related, and family-related variables were collected before model development. The study population consisted of children and adolescents who were assessed for suspected functional abdominal pain disorders during the study period. The study protocol was approved by the Ethics Committee of The Second Affiliated Hospital & Yuying Children’s Hospital of Wenzhou Medical University (Approval number: Lunshen 2026-K-226-01). The requirement for informed consent was waived by the Ethics Committee because of the retrospective nature of the study and the use of de-identified routinely collected data. All data were anonymized before analysis, and the study was conducted in accordance with the Declaration of Helsinki.

Eligible participants were patients aged 7–18 years who fulfilled the diagnostic criteria for functional abdominal pain disorders and had complete information on the outcome assessment and sufficient information on key candidate predictors. Patients were excluded if they did not meet Rome IV diagnostic criteria for functional abdominal pain disorders, lacked standardized anxiety assessment, had received previous psychological or psychiatric treatment before the abdominal pain assessment, had substantial missingness in key clinical, psychosocial, or family-related predictor data, or met other prespecified exclusion criteria. A total of 629 eligible patients were included in the final analysis.

The included patients were randomly divided into a training cohort and an internal validation cohort at a 7:3 ratio using stratified random sampling according to anxiety status. The training cohort included 440 patients, consisting of 147 patients with comorbid anxiety disorder and 293 patients without anxiety disorder. The validation cohort included 189 patients, consisting of 63 patients with comorbid anxiety disorder and 126 patients without anxiety disorder (**Figure 1**). This stratified allocation was used to preserve a comparable outcome distribution between the training and validation datasets. The training cohort was used for predictor preprocessing, feature selection, hyperparameter tuning, model development, probability calibration, and threshold determination, whereas the validation cohort was reserved exclusively for final model evaluation.

**Figure 1:**
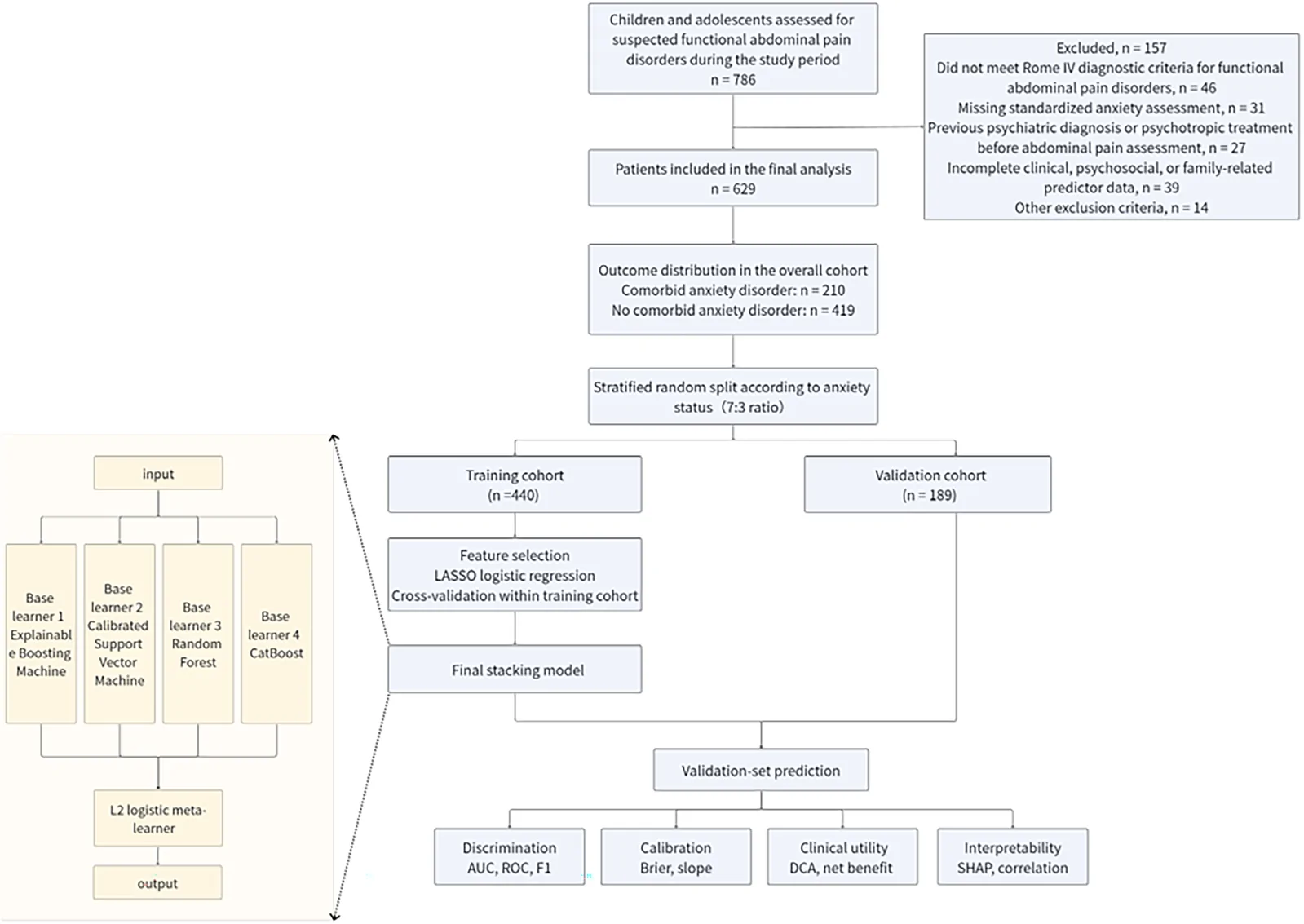
Study flowchart and stacking model development pipeline. The full four-learner stacking model was prespecified before validation-set evaluation. The validation cohort was not used for feature selection, hyperparameter tuning, base-learner selection, meta-learner training, threshold determination, or model-combination selection.

### 2.2 Diagnostic criteria and outcome definition

Functional abdominal pain disorders were diagnosed according to the Rome IV criteria. The diagnostic categories included functional dyspepsia, irritable bowel syndrome, abdominal migraine, and functional abdominal pain not otherwise specified. The diagnosis was established by experienced pediatric gastroenterologists based on clinical history, symptom characteristics, physical examination, and available auxiliary examinations. Patients with evidence of organic gastrointestinal disease, inflammatory disease, metabolic disease, infectious disease, structural abnormalities, or other identifiable organic causes of abdominal pain were not considered to have functional abdominal pain disorders and were excluded from the modeling cohort.

The primary outcome was the presence of comorbid anxiety disorder at baseline. Anxiety symptoms were first screened using the Chinese version of the Screen for Child Anxiety Related Emotional Disorders (SCARED) (17). The SCARED is a 41-item self-report instrument for assessing anxiety symptoms in children and adolescents. Each item is scored on a 3-point scale, with 0 indicating “not true or hardly ever true,” 1 indicating “somewhat true or sometimes true,” and 2 indicating “very true or often true.” The total score ranges from 0 to 82, with higher scores indicating more severe anxiety symptoms. A total SCARED score of 25 or higher was considered a positive screening result for clinically significant anxiety symptoms. For younger children, SCARED responses were completed with age-appropriate explanation when necessary, and parent-reported information was considered during the subsequent clinical evaluation.

The SCARED includes five anxiety-related dimensions: panic/somatic symptoms, generalized anxiety, separation anxiety, social anxiety, and school avoidance. Commonly used subscale thresholds were 7 or higher for panic/somatic symptoms, 9 or higher for generalized anxiety, 5 or higher for separation anxiety, 8 or higher for social anxiety, and 3 or higher for school avoidance. These SCARED total and subscale scores were used only to support clinical assessment and characterize the anxiety symptom profile. They were not used as predictors in any machine-learning model.

The final diagnosis of comorbid anxiety disorder was determined by trained child psychiatrists or clinical psychologists according to the Diagnostic and Statistical Manual of Mental Disorders, Fifth Edition (DSM-5) criteria. Children and adolescents were classified into the anxiety group if they met DSM-5 diagnostic criteria for at least one anxiety disorder during the baseline clinical evaluation, including generalized anxiety disorder, separation anxiety disorder, social anxiety disorder, panic disorder, specific phobia, or other clinically defined anxiety disorders. Participants who did not meet DSM-5 diagnostic criteria for any anxiety disorder were classified into the non-anxiety group. Therefore, SCARED screening results were used as an auxiliary assessment rather than as the sole diagnostic basis for anxiety disorder.

The anxiety assessment was completed before model development and before any feature selection, hyperparameter tuning, model training, probability calibration, or threshold determination. Clinicians responsible for anxiety diagnosis were not involved in model development. When diagnostic uncertainty occurred, cases were reviewed by a senior child psychiatrist, and the final classification was determined by clinical consensus according to predefined diagnostic procedures.

To minimize information leakage, variables that directly represented the diagnostic outcome or were obtained after anxiety diagnosis were not included as candidate predictors. Specifically, SCARED total scores, SCARED subscale scores, clinician diagnostic conclusions, psychiatric interview results, and post-diagnostic mental health treatment variables were excluded from all machine-learning models. If school avoidance was included as a candidate predictor, it referred only to independently collected school-functioning information, such as structured clinical information, parent-reported school attendance problems, or school-related functional impairment, and not to the SCARED-derived school avoidance subscale. This strategy was used to ensure that the model estimated the probability of comorbid anxiety disorder from routinely obtainable demographic, abdominal pain-related, psychosocial, school-related, and family-level information rather than reproducing the anxiety diagnostic instrument itself.

### 2.3 Candidate predictors and clinical, psychosocial, and family assessments

Candidate predictors were selected based on clinical relevance, prior evidence regarding the gut–brain interaction in functional abdominal pain disorders, and the availability of variables before outcome classification. Demographic variables included age, sex, body mass index, residence, and only-child status. Clinical variables related to functional abdominal pain disorders included symptom duration, abdominal pain frequency, abdominal pain intensity assessed using a numerical rating scale, morning abdominal pain, night-time awakening due to pain, abdominal pain temporally associated with stressful events, Functional Disability Inventory score, school absence, previous emergency visit for abdominal pain, previous gastrointestinal medication use, and functional abdominal pain disorder subtype.

School absence was defined as the number of school days actually missed during the preceding month and was recorded as days per month. School avoidance was assessed separately as a binary variable (0 = no, 1 = yes). During the same assessment period, specific school-attendance behaviors were determined based on parent-provided information and clinical records. School avoidance was coded as present when the child was documented as refusing to attend school as scheduled, refusing to enter or remain at school or in the classroom after arrival, or requesting to leave school early in the absence of an evident acute worsening of illness, a medical visit, or another objective reason requiring departure. Children in whom such behaviors were not identified through inquiry or clinical assessment were coded as 0. This variable reflected the presence rather than the frequency of school-attendance avoidance behavior, was classified independently of the actual number of school days missed, and was assessed independently of the SCARED school-avoidance subscale. Associated somatic symptoms and comorbid conditions, including constipation, diarrhea, nausea, abdominal bloating, headache or migraine, and allergic diseases, were also recorded.

Child psychosocial and school-related variables were assessed using standardized scales and structured clinical information. Sleep disturbance was evaluated using the Sleep Disturbance Scale for Children, with sleep disturbance defined according to the prespecified threshold. Somatic symptom burden was assessed using the Children’s Somatic Symptoms Inventory-24. Additional child-level psychosocial variables included pain catastrophizing score, depressive symptom score, school avoidance, peer relationship difficulties, and recent academic stress. These variables were included because anxiety in children and adolescents with functional abdominal pain disorders may be associated not only with pain severity but also with sleep problems, somatic amplification, school impairment, and psychosocial stress.

Parent and family characteristics were also collected as candidate predictors. These included primary caregiver, single-parent family status, left-behind child status, maternal and paternal education, monthly household income relative to the local median, parental marital conflict, parental anxiety symptoms assessed using the Generalized Anxiety Disorder-7 scale, parental depressive symptoms, parental history of anxiety or depression, family history of psychiatric disorders, parental pain catastrophizing score, overprotective parenting style, high parental health-care seeking tendency, and family history of recurrent abdominal pain. All predictors were measured before model training, and post-diagnostic variables were excluded. Continuous variables were retained in their original form unless transformation was prespecified during preprocessing, whereas categorical variables were coded according to clinically meaningful categories.

### 2.4 Data splitting, preprocessing, missing data, and leakage prevention

After eligibility assessment, the final analytic cohort was divided into a training cohort and an internal validation cohort at a 7:3 ratio using stratified random sampling according to anxiety status. Stratification was used to preserve a comparable event proportion between patients with and without comorbid anxiety disorder in the two datasets. The split was performed before any model-development procedure. The training cohort was used for all procedures related to model construction, including imputation, preprocessing, feature selection, hyperparameter tuning, probability calibration, threshold determination, and meta-learner training. The validation cohort was kept independent from model development and was used only for final performance reporting of prespecified models and exploratory sensitivity analyses; it was not used for selecting the final model, tuning model parameters, choosing learner combinations, calibrating probabilities, or determining classification thresholds.

Missing data were handled according to prespecified rules. Patients with missing outcome assessment or missing information essential for defining functional abdominal pain disorders or anxiety status were excluded before the analytic cohort was finalized. Patients with substantial missingness in key clinical, psychosocial, or family-related predictors were also excluded according to the predefined eligibility criteria. Overall, 248 of 27,676 candidate-predictor observations (0.90%) were missing before imputation, and the maximum variable-specific missingness was 3.2%. No outcome values were imputed. Variable-specific missingness and the corresponding imputation methods are reported in **Supplementary Table S1**. For candidate predictors with limited residual missingness, single imputation was embedded within the modeling pipeline. Continuous variables were imputed using the median estimated exclusively from the corresponding training data, whereas categorical variables were imputed using the most frequent category. During cross-validation, all imputation parameters were estimated only from the corresponding training fold and then applied to the held-out fold. For validation-set prediction, imputation rules estimated from the full training cohort were applied unchanged to the validation cohort. This ensured that no information from the held-out folds or validation cohort contributed to the estimation of imputation parameters.

All preprocessing steps were embedded within the training pipeline to prevent information leakage. Right-skewed variables, including symptom duration, abdominal pain frequency, and school absence, were transformed using log2(1 + x). Categorical predictors were encoded using coding rules learned from the corresponding training fold. Continuous predictors were standardized when required by the algorithm, particularly for support vector machine modeling, whereas tree-based models were fitted using the processed feature matrix without unnecessary scaling. In each cross-validation split, imputation, categorical encoding, scaling, LASSO-based feature selection, hyperparameter tuning, model fitting, probability calibration, and threshold determination were performed using only the training fold. The held-out fold was used exclusively to generate out-of-fold predicted probabilities. No information from the held-out fold or the validation cohort was used in any preprocessing, feature-selection, model-tuning, calibration, threshold-selection, or meta-learning step.

### 2.5 Feature selection and development of base learners

Feature selection was performed using least absolute shrinkage and selection operator logistic regression within the training cohort. Candidate predictors were selected *a priori* based on clinical relevance, biological plausibility, and availability before anxiety outcome classification. For out-of-fold prediction, LASSO feature selection was repeated within each training fold, and the LASSO penalty parameter was selected using cross-validation within that fold. Predictors with non-zero coefficients at the selected λ were retained for model fitting in the corresponding fold. This design ensured that feature selection did not use information from the held-out fold. For final validation-set prediction, the final predictor set was determined using the full training cohort only. LASSO was used as a dimensionality-reduction step to reduce overfitting and improve model parsimony, rather than as a method for causal variable discovery.

Four prespecified base learners were developed using the selected predictors: explainable boosting machine, calibrated support vector machine, random forest, and CatBoost. These algorithms were chosen to provide complementary modeling characteristics. The explainable boosting machine was included to capture non-linear additive effects while preserving model interpretability. The support vector machine was included as a margin-based classifier requiring standardized predictors and probability calibration. Random forest was used as a bagging-based tree ensemble capable of modeling non-linear associations and interactions. CatBoost was included as a regularized gradient boosting model suitable for tabular clinical data and complex predictor relationships.

Hyperparameters for each base learner were optimized within the training cohort. During out-of-fold prediction, hyperparameter tuning was performed within each training fold, using inner resampling when applicable. After imputation, encoding, scaling when required, LASSO-based feature selection, hyperparameter tuning, model fitting, and probability calibration were completed within the training fold, the corresponding held-out fold was used only to generate predicted probabilities. These fold-wise held-out predictions were concatenated across all folds to obtain out-of-fold predicted probabilities for each base learner. After the modeling procedures and hyperparameters were finalized within the training data, each base learner was refitted on the full training cohort according to the finalized training-derived pipeline before being applied to the validation cohort.

### 2.6 Stacking ensemble construction, calibration, and threshold determination

The stacking ensemble was constructed using out-of-fold predicted probabilities generated by the four base learners in the training cohort. For each patient in the training cohort, the meta-features consisted only of predicted probabilities obtained when that patient belonged to the held-out fold. The out-of-fold probabilities from the explainable boosting machine, support vector machine, random forest, and CatBoost models were then used as inputs to train an L2-penalized logistic regression meta-learner. This meta-learner combined the base-learner outputs and estimated the final probability of comorbid anxiety disorder. The full four-learner stacking model was prespecified before validation-set evaluation.

The use of out-of-fold probabilities prevented the meta-learner from being trained on overly optimistic in-sample predictions from the base learners. For validation-set prediction, the finalized base learners were first refitted on the entire training cohort using the full training-derived pipeline, including imputation, preprocessing, selected predictors, tuned hyperparameters, and calibration procedures. These refitted base learners were then applied to the independent validation cohort to generate four validation-set predicted probabilities for each patient. The resulting validation-set base-learner probabilities were passed to the already trained L2-penalized logistic meta-learner to obtain the final stacking predicted risks. Candidate smaller stacking combinations were assessed only as exploratory sensitivity analyses and were not used to select the final model.

Probability calibration was performed within the training pipeline when required by the model structure. For models requiring calibrated probabilities, calibration parameters were estimated only from the corresponding training fold during out-of-fold prediction or from the full training cohort during final refitting. Classification thresholds were determined exclusively in the training cohort using the Youden index based on training-derived predicted probabilities. The selected thresholds were fixed before validation-set evaluation and were applied unchanged to the validation cohort. No calibration parameter, probability threshold, learner weight, or stacking combination was re-estimated using validation-set information. Penalized meta-learner coefficients were reported descriptively to indicate the relative contribution of each base learner and were not interpreted as clinical odds ratios or inferential effect estimates.

### 2.7 Model evaluation, statistical analysis, interpretability, and ethics

Baseline characteristics were summarized for the overall cohort, training cohort, and validation cohort. Continuous variables were reported as mean ± standard deviation or median with interquartile range according to their distribution, and categorical variables were reported as counts and percentages. Balance between the training and validation cohorts was assessed using standardized mean differences rather than hypothesis testing. Descriptive multivariable logistic regression was used to examine clinically interpretable associations between selected predictors and comorbid anxiety disorder, with adjusted odds ratios, 95% confidence intervals, and P values reported. These regression analyses were considered descriptive and were not interpreted as evidence of causality. P values from these analyses were not used for predictor screening in the machine learning pipeline.

Model performance was assessed using out-of-fold estimates in the training cohort and final estimates in the held-out validation cohort. Discrimination was evaluated using the area under the receiver operating characteristic curve. Threshold-dependent classification performance was assessed using accuracy, sensitivity, specificity, positive predictive value, negative predictive value, F1 score, and Cohen’s kappa, as appropriate. Probability estimation was evaluated using the Brier score and calibration curves. Clinical utility was examined using decision curve analysis by comparing the net benefit of the model-guided strategy with treat-all and treat-none strategies across a range of threshold probabilities. Confidence intervals for performance metrics were estimated using resampling-based methods.

Learner diversity was assessed using predictions generated exclusively through out-of-fold cross-validation in the training cohort. For each pair of base learners, we calculated the Pearson correlation between predicted probabilities, classification disagreement, Cohen’s kappa, double-fault rate, Yule’s Q, and entropy-based diversity. For stacking combinations containing three or more learners, pairwise correlation, disagreement, double-fault rate, and entropy-based diversity were calculated for all possible learner pairs and summarized using the mean pairwise value.

For descriptive radar-plot visualization, both predictive-performance and learner-diversity measures were derived exclusively from out-of-fold predictions in the training cohort and were rescaled to a common range from 0 to 1. For measures in which higher values indicated a more favorable profile, including the area under the receiver operating characteristic curve, F1 score, prediction disagreement, and entropy-based diversity, min–max normalization was performed as follows:

z = (x − xmin)/(xmax − xmin).

For measures in which lower values indicated a more favorable profile, including the Brier score, double-fault rate, and mean pairwise correlation between predicted probabilities, reverse min–max normalization was performed as follows:

z = (xmax − x)/(xmax − xmin).

Accordingly, larger values on all radar-plot axes represented a more favorable descriptive profile. Lower probability correlation was interpreted as indicating greater diversity between learners rather than better predictive accuracy.

Because min–max normalization depends on the candidate combinations included in the comparison, and because no prespecified weighting scheme was used to combine the individual dimensions, the radar plots were interpreted as descriptive visualizations only. Neither polygon area nor apparent balance across axes was treated as a formal composite score, and the radar-plot analysis was not used for model selection or for statistical claims of superiority. The complete four-learner stacking model had been prespecified before validation-set evaluation. Raw values for all performance and diversity measures were additionally reported in the Supplementary Materials.

Model interpretability was assessed using SHAP analysis applied to the complete stacking pipeline. SHAP values were calculated on the log-odds scale of the final stacking model output rather than directly on the probability scale. The original clinical predictors were used as explanatory features. Positive SHAP values indicated that a predictor shifted the model output toward a higher estimated probability of comorbid anxiety disorder, whereas negative SHAP values indicated a shift toward a lower estimated probability. SHAP summary plots, feature-importance plots, and individual-level explanation plots were generated to characterize global and patient-level contributors to the model output.

## 3 Results

### 3.1 Study population and baseline characteristics

Between December 2021 and December 2025, a total of 629 eligible children and adolescents with functional abdominal pain disorders were included in the final analysis. Using stratified random sampling, patients were allocated at a 7:3 ratio into a training cohort of 440 patients and a validation cohort of 189 patients. The overall prevalence of comorbid anxiety disorder was 33.4%, with 210 affected patients among the full cohort. The distribution of anxiety status was highly consistent between cohorts: the training cohort included 147 patients with anxiety disorder and 293 patients without anxiety disorder, while the validation cohort included 63 patients with anxiety disorder and 126 patients without anxiety disorder. This allocation maintained a comparable event proportion between the training and validation datasets (**Table 1**).

**Table 1:** Baseline characteristics of the training and validation cohorts.

Characteristic	Overall cohort (n = 629)	Training cohort (n = 440)	Validation cohort (n = 189)	SMD
Child demographic characteristics				
Age, years	11.8 ± 2.9	11.7 ± 2.8	12.0 ± 3.0	0.10
Age 7–12 years	386 (61.4)	274 (62.3)	112 (59.3)	0.06
Age 13–18 years	243 (38.6)	166 (37.7)	77 (40.7)	0.06
Male sex	286 (45.5)	199 (45.2)	87 (46.0)	0.02
Female sex	343 (54.5)	241 (54.8)	102 (54.0)	0.02
Body mass index, kg/m²	18.9 ± 3.2	18.8 ± 3.1	19.1 ± 3.3	0.09
Urban residence	397 (63.1)	276 (62.7)	121 (64.0)	0.03
Only child	291 (46.3)	205 (46.6)	86 (45.5)	0.02
Clinical characteristics of functional abdominal pain disorders				
Symptom duration, months	8.0 (4.0–15.0)	8.0 (4.0–14.0)	9.0 (4.0–16.0)	0.08
Abdominal pain frequency, episodes/week	4.0 (2.0–6.0)	4.0 (2.0–6.0)	4.0 (2.0–7.0)	0.05
Abdominal pain intensity, NRS score	5.4 ± 1.8	5.3 ± 1.8	5.5 ± 1.9	0.11
Morning abdominal pain	176 (28.0)	121 (27.5)	55 (29.1)	0.04
Night-time awakening due to pain	93 (14.8)	64 (14.5)	29 (15.3)	0.02
Abdominal pain temporally associated with stressful events	201 (32.0)	139 (31.6)	62 (32.8)	0.03
Functional Disability Inventory score	14.6 ± 7.8	14.4 ± 7.6	15.0 ± 8.1	0.08
School absence, days/month	2.0 (0.0–5.0)	2.0 (0.0–5.0)	2.0 (0.0–6.0)	0.07
Previous emergency visit for abdominal pain	88 (14.0)	60 (13.6)	28 (14.8)	0.03
Previous gastrointestinal medication use	246 (39.1)	171 (38.9)	75 (39.7)	0.02
Functional dyspepsia	198 (31.5)	139 (31.6)	59 (31.2)	0.01
Irritable bowel syndrome	176 (28.0)	121 (27.5)	55 (29.1)	0.04
Abdominal migraine	74 (11.8)	51 (11.6)	23 (12.2)	0.02
Functional abdominal pain, not otherwise specified	181 (28.8)	129 (29.3)	52 (27.5)	0.04
Constipation	167 (26.6)	116 (26.4)	51 (27.0)	0.01
Diarrhea	122 (19.4)	87 (19.8)	35 (18.5)	0.03
Nausea	156 (24.8)	106 (24.1)	50 (26.5)	0.05
Abdominal bloating	214 (34.0)	151 (34.3)	63 (33.3)	0.02
Headache or migraine	83 (13.2)	57 (13.0)	26 (13.8)	0.02
Allergic diseases	141 (22.4)	99 (22.5)	42 (22.2)	0.01
Child psychosocial and school-related characteristics				
Sleep disturbance, SDSC >39	189 (30.0)	134 (30.5)	55 (29.1)	0.03
SDSC total score	35.6 ± 8.4	35.4 ± 8.3	36.0 ± 8.6	0.07
School avoidance	136 (21.6)	93 (21.1)	43 (22.8)	0.04
Peer relationship difficulties	112 (17.8)	79 (18.0)	33 (17.5)	0.01
Recent academic stress	221 (35.1)	156 (35.5)	65 (34.4)	0.02
CSI-24 score	22.4 ± 10.6	22.1 ± 10.4	23.0 ± 11.0	0.08
Child pain catastrophizing score	19.6 ± 9.1	19.4 ± 8.9	20.1 ± 9.5	0.08
Child depressive symptom score	11.8 ± 6.2	11.6 ± 6.1	12.2 ± 6.4	0.10
Comorbid anxiety disorder	210 (33.4)	147 (33.4)	63 (33.3)	0.00
Parent and family characteristics				
Mother as primary caregiver	465 (73.9)	326 (74.1)	139 (73.5)	0.01
Father as primary caregiver	61 (9.7)	42 (9.5)	19 (10.1)	0.02
Grandparent or other primary caregiver	103 (16.4)	72 (16.4)	31 (16.4)	0.00
Single-parent family	72 (11.4)	51 (11.6)	21 (11.1)	0.02
Left-behind child status	68 (10.8)	46 (10.5)	22 (11.6)	0.04
Maternal education of college or above	312 (49.6)	216 (49.1)	96 (50.8)	0.03
Paternal education of college or above	295 (46.9)	207 (47.0)	88 (46.6)	0.01
Monthly household income above local median	281 (44.7)	195 (44.3)	86 (45.5)	0.02
Parental marital conflict	96 (15.3)	66 (15.0)	30 (15.9)	0.02
Parental anxiety symptoms, GAD-7 ≥10	148 (23.5)	103 (23.4)	45 (23.8)	0.01
Parental GAD-7 score	5.0 (2.0–9.0)	5.0 (2.0–9.0)	5.0 (2.0–9.0)	0.03
Parental depressive symptoms	116 (18.4)	80 (18.2)	36 (19.0)	0.02
Parental history of anxiety or depression	118 (18.8)	82 (18.6)	36 (19.0)	0.01
Family history of psychiatric disorders	76 (12.1)	52 (11.8)	24 (12.7)	0.03
Parental pain catastrophizing score	18.7 ± 8.9	18.5 ± 8.7	19.2 ± 9.2	0.08
Overprotective parenting style	154 (24.5)	107 (24.3)	47 (24.9)	0.01
High parental health-care seeking tendency	131 (20.8)	90 (20.5)	41 (21.7)	0.03
Family history of recurrent abdominal pain	139 (22.1)	96 (21.8)	43 (22.8)	0.02

Data are presented as mean ± SD, median (IQR), or n (%). SMDs compare the training and validation cohorts. NRS, numerical rating scale; FDI, Functional Disability Inventory; SDSC, Sleep Disturbance Scale for Children; CSI-24, Children’s Somatic Symptoms Inventory-24; GAD-7, Generalized Anxiety Disorder-7.

Baseline characteristics were largely comparable between the two cohorts across demographic, clinical, psychosocial, and family related domains. The mean age was 11.7 ± 2.8 years in the training cohort and 12.0 ± 3.0 years in the validation cohort, with female patients accounting for 54.8% and 54.0%, respectively. Median symptom duration was 8.0 months in the training cohort and 9.0 months in the validation cohort, while the median abdominal pain frequency was 4.0 episodes per week in both cohorts. The distributions of functional dyspepsia, irritable bowel syndrome, abdominal migraine, and functional abdominal pain not otherwise specified were similar between datasets. Psychosocial and family related variables, including SDSC total score, CSI 24 score, parental GAD 7 score, parental marital conflict, and family history of psychiatric disorders, also showed no substantial imbalance. Most standardized mean differences were small, supporting adequate baseline comparability after stratified allocation, although abdominal pain intensity showed a slightly higher standardized mean difference of 0.11 (**Table 1**). Before model development, all predictor preprocessing, LASSO-based feature selection, hyperparameter tuning, probability calibration, and classification-threshold determination were performed exclusively within the training cohort, whereas the validation cohort was reserved only for final model evaluation (**Figure 1**). Residual missingness in the final analytic cohort was low. Overall, 248 of 27,676 candidate-predictor observations (0.90%) were missing before imputation, with a maximum variable-specific missingness of 3.2%. No outcome values were missing. Variable-specific missingness and the corresponding imputation methods are reported in **Supplementary Table S1**.

### 3.2 Predictor selection and multivariable associations with comorbid anxiety

Multivariable logistic regression identified clinical, psychosocial, and family related factors associated with comorbid anxiety disorder in children and adolescents with functional abdominal pain disorders. Older age showed a modest association with anxiety, with an adjusted odds ratio of 1.07 per one year increase, 95% CI 1.00 to 1.15, P = 0.041. Longer symptom duration and higher abdominal pain frequency remained associated with anxiety after log2 transformation, with adjusted odds ratios of 1.31, 95% CI 1.08 to 1.59, P = 0.006, and 1.36, 95% CI 1.10 to 1.68, P = 0.004, respectively. Among pain related features, night time awakening due to pain was associated with an adjusted odds ratio of 1.55, 95% CI 1.01 to 2.39, P = 0.045, while abdominal pain temporally associated with stressful events showed an adjusted odds ratio of 1.86, 95% CI 1.28 to 2.70, P = 0.001 (**Table 2**).

**Table 2:** Multivariable logistic regression analysis for comorbid anxiety disorder in children and adolescents with functional abdominal pain disorders.

Variable	Coding/transformation	β coefficient	Adjusted OR	95% CI	P value
Age, years	Per 1-year increase	0.07	1.07	1.00–1.15	0.041
Symptom duration, months	log2(1 + x)	0.27	1.31	1.08–1.59	0.006
Abdominal pain frequency, episodes/week	log2(1 + x)	0.31	1.36	1.10–1.68	0.004
Night-time awakening due to pain	Yes vs no	0.44	1.55	1.01–2.39	0.045
Abdominal pain temporally associated with stressful events	Yes vs no	0.62	1.86	1.28–2.70	0.001
SDSC total score	Per 5-point increase	0.17	1.18	1.07–1.31	0.001
CSI-24 score	Per 5-point increase	0.23	1.26	1.13–1.41	<0.001
Parental GAD-7 score	Per 5-point increase	0.38	1.46	1.19–1.79	<0.001
School absence, days/month	log2(1 + x)	0.25	1.28	1.04–1.58	0.020

The outcome was comorbid anxiety disorder. Right-skewed variables were transformed using log2(1+x). SDSC, CSI-24, and parental GAD-7 scores were modeled per 5-point increase. SCARED scores and post-diagnostic variables were excluded to minimize information leakage.

Psychosocial and family level variables also contributed to the anxiety risk profile. Each 5 point increase in SDSC total score was associated with an adjusted odds ratio of 1.18, 95% CI 1.07 to 1.31, P = 0.001, and each 5 point increase in CSI 24 score was associated with an adjusted odds ratio of 1.26, 95% CI 1.13 to 1.41, P < 0.001. Parental GAD 7 score showed the strongest family related association, with an adjusted odds ratio of 1.46 per 5 point increase, 95% CI 1.19 to 1.79, P < 0.001. School absence also remained associated with anxiety after log2 transformation, with an adjusted odds ratio of 1.28, 95% CI 1.04 to 1.58, P = 0.020. LASSO regression was then performed in the training cohort for feature selection. The coefficient profile showed progressive shrinkage of candidate predictors as the penalty parameter increased, and cross validation identified the optimal λ for balancing model complexity and predictive performance. Predictors with non zero coefficients at the selected λ were retained for subsequent model development (**Figures 2A, B**).

**Figure 2:**
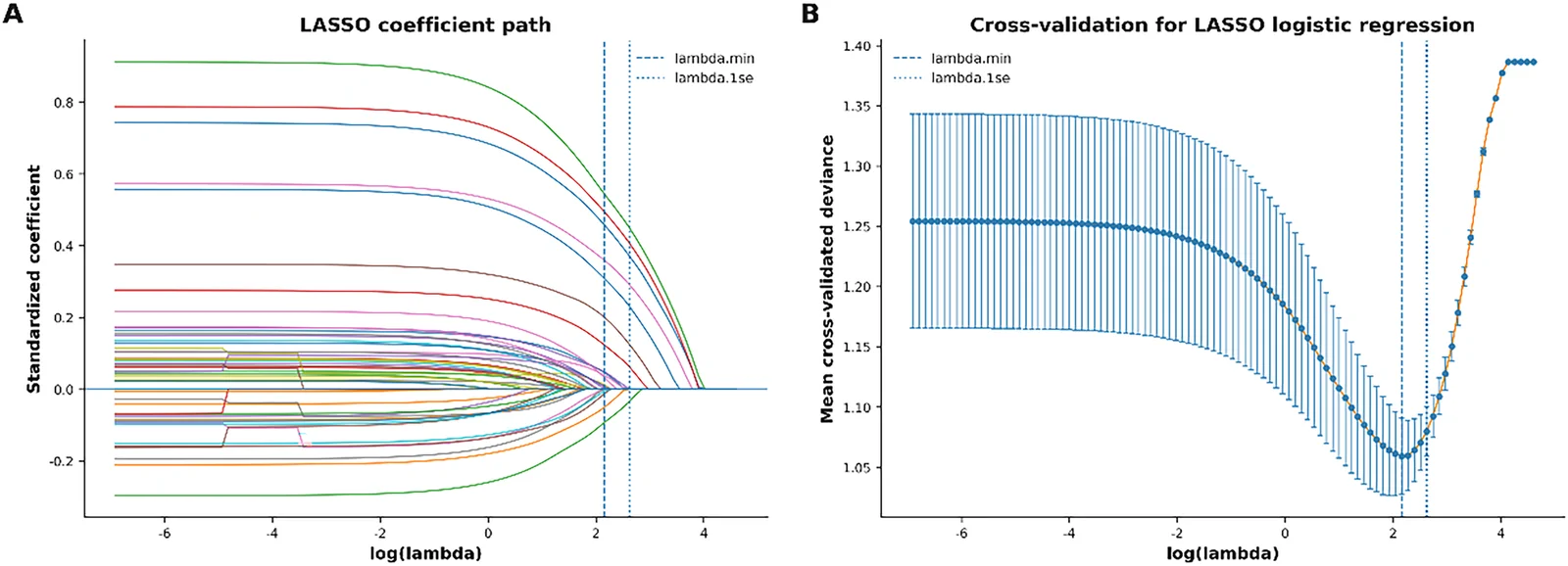
LASSO feature selection and cross-validation. LASSO regression was used for feature selection. The coefficient profile shows changes in predictor coefficients across different values of the penalty parameter λ. Cross-validation was used to identify the optimal λ, balancing model complexity and predictive performance. Predictors with non-zero coefficients at the selected λ were retained for subsequent model development. LASSO, least absolute shrinkage and selection operator; CV, cross-validation.

### 3.3 Model comparison, learner diversity, and stacking performance

Candidate models were compared using discrimination, classification, and calibration-related metrics. In the training cohort, out-of-fold cross-validation showed that the prespecified full stacking model achieved an AUC of 0.831, 95% CI 0.789 to 0.868, accuracy of 0.768, 95% CI 0.726 to 0.806, sensitivity of 0.735, 95% CI 0.658 to 0.800, specificity of 0.785, 95% CI 0.734 to 0.830, and a Brier score of 0.151, 95% CI 0.135 to 0.169. Among the individual base learners, CatBoost showed the strongest out-of-fold discrimination, followed by Random Forest, EBM, and SVM, with AUC values of 0.808, 0.786, 0.776, and 0.762, respectively. Accuracy, sensitivity, and Cohen’s kappa further demonstrated heterogeneous classification performance across individual learners, supporting the comparison of their outputs before ensemble construction (**Figure 3**; **Supplementary Table S2**).

**Figure 3:**
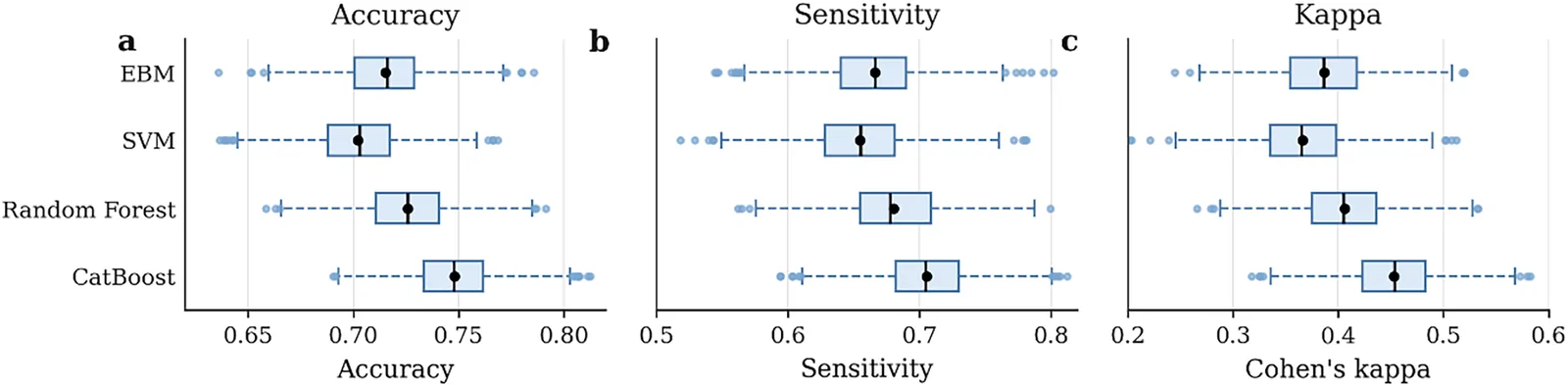
Comparison of classification performance across candidate machine learning models. **(a)** Accuracy. **(b)** Sensitivity. **(c)** Cohen's kappa. The boxplots illustrate the distribution of each performance metric across the EBM, SVM, Random Forest, and CatBoost models.

In the L2-penalized logistic meta-learner, all four base-learner predicted probabilities had positive coefficients and contributed to the final stacked prediction. CatBoost showed the largest penalized coefficient, followed by EBM, Random Forest, and SVM, suggesting the greatest relative contribution of CatBoost among the four base learners. These coefficients were interpreted descriptively as meta-learner weights rather than as inferential clinical effect estimates (**Supplementary Table S3**).

Pairwise diversity analyses showed that the base learners provided complementary predictions rather than identical outputs. Correlations of out-of-fold predicted probabilities ranged from 0.55 for EBM versus CatBoost to 0.68 for Random Forest versus CatBoost, while prediction disagreement ranged from 0.24 to 0.33 and double-fault rates ranged from 0.103 to 0.150 (**Supplementary Figure S1**; **Supplementary Table S4**).

In the validation cohort, the prespecified full stacking model integrating EBM, SVM, Random Forest, and CatBoost showed the highest numerical internal-validation performance among the individual base learners and the final stacking model, with an AUC of 0.846, 95% CI 0.789 to 0.894, accuracy of 0.788, 95% CI 0.725 to 0.841, sensitivity of 0.762, 95% CI 0.644 to 0.850, specificity of 0.802, 95% CI 0.723 to 0.862, positive predictive value of 0.658, 95% CI 0.543 to 0.756, negative predictive value of 0.871, 95% CI 0.798 to 0.920, F1 score of 0.706, 95% CI 0.611 to 0.788, and Brier score of 0.146, 95% CI 0.126 to 0.169. The full four-learner stacking model had been prespecified as the final model before validation-set evaluation. Comparisons with smaller stacking combinations were performed only as exploratory analyses based on out-of-fold training predictions and were not used for final model selection (**Figure 4**; **Supplementary Table S5**).

**Figure 4:**
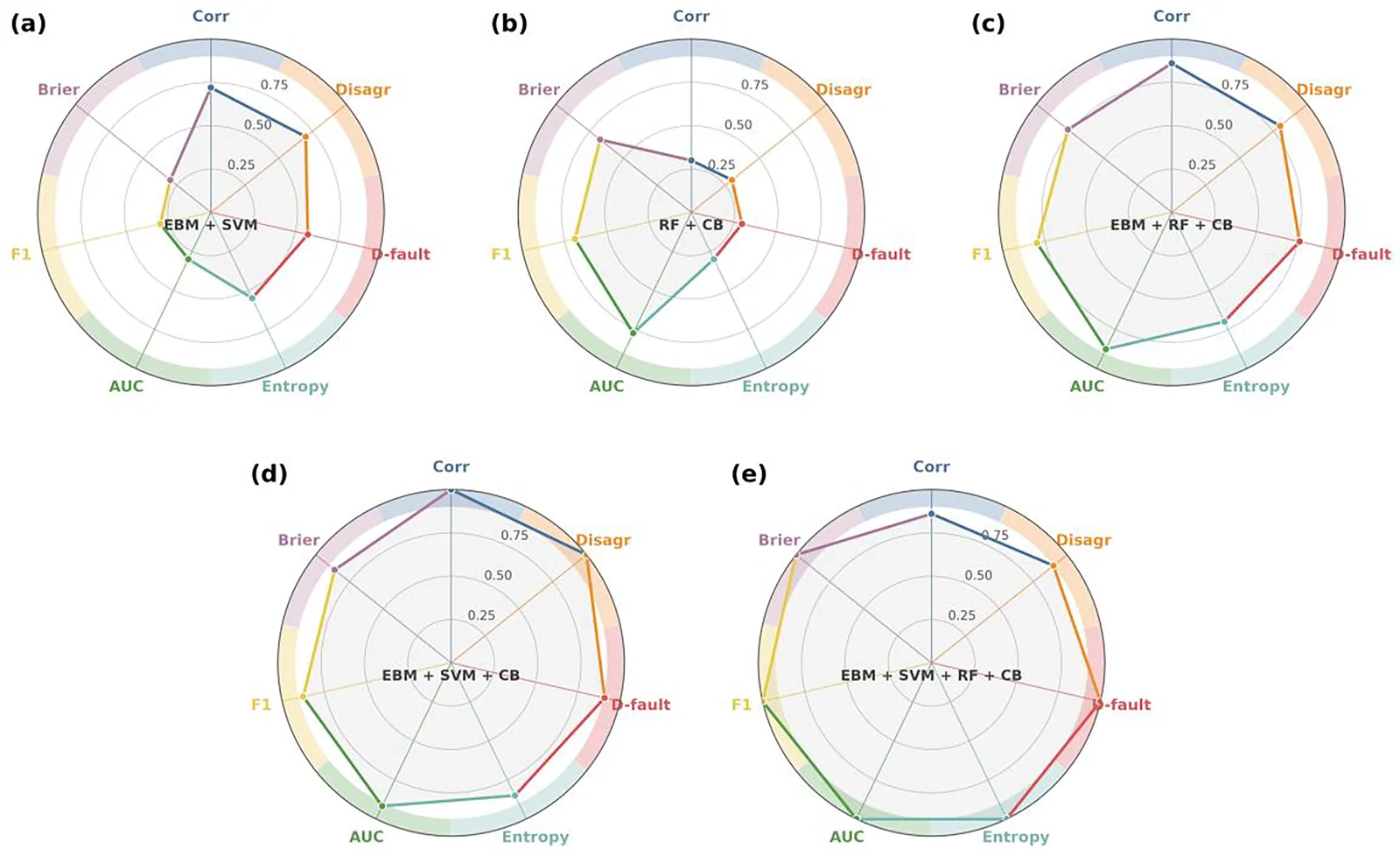
Descriptive out-of-fold learner-diversity and predictive-performance profiles of candidate stacking combinations. All measures were derived from out-of-fold predictions in the training cohort. AUC, F1 score, disagreement, and entropy were normalized so that higher values indicated a more favorable profile, whereas Brier score, double-fault rate, and probability correlation were reverse-normalized. For combinations with more than two learners, diversity measures were averaged across all learner pairs. The radar plots were descriptive only and were not used for model selection. **(a)** EBM + SVM; **(b)** Random Forest + CatBoost; **(c)** EBM + Random Forest + CatBoost; **(d)** EBM + SVM + CatBoost; **(e)** EBM + SVM + Random Forest + CatBoost.

Radar-based learner-diversity and out-of-fold performance profiles were descriptively examined for representative candidate stacking combinations using out-of-fold predictions from the training cohort. All displayed metrics were oriented so that larger normalized values indicated a more favorable profile. Mean pairwise probability correlation, mean pairwise double-fault rate, and Brier score were reverse-normalized, whereas AUC, F1 score, mean pairwise disagreement, and entropy-based diversity were normalized in the original direction.

The candidate combinations showed different trade-offs across learner-diversity and out-of-fold performance measures. The full four-learner combination showed the highest entropy-based diversity, whereas the EBM + SVM + CatBoost combination showed the lowest mean pairwise probability correlation, the lowest mean pairwise double-fault rate, the highest out-of-fold AUC, and the lowest out-of-fold Brier score. However, no candidate combination was uniformly superior across all evaluated dimensions. Because min–max normalization depends on the set of candidate combinations included in the comparison and no prespecified weighting scheme was used to combine the different metrics, the radar profiles were interpreted as descriptive visualizations only. They were not treated as composite scores and were not used to establish statistical superiority or to select the final stacking model.

### 3.4 Discrimination, clinical utility, and calibration of the final stacking model

The discriminative performance of the final stacking model was further evaluated using ROC analysis in both the training and validation cohorts. The ROC curves showed consistent separation between patients with and without comorbid anxiety disorder, and the stacking model demonstrated numerically higher discrimination than the individual base learners. Although the validation AUC was slightly higher than the out-of-fold training AUC, this difference should not be interpreted as evidence of superior generalization. Given the moderate sample size of the validation cohort and the single random split design, the higher validation estimate may have resulted from sampling variability or a relatively less difficult validation subset. Accordingly, the validation results indicate acceptable internal-validation performance, while external validation remains necessary to assess transportability.

In the validation cohort, the stacking model achieved the highest AUC among all candidate models, with an AUC of 0.846, 95% CI 0.789 to 0.894. Its threshold-dependent classification performance was also balanced, with an accuracy of 0.788, sensitivity of 0.762, specificity of 0.802, positive predictive value of 0.658, negative predictive value of 0.871, and F1 score of 0.706. These findings were generally consistent with the ROC patterns observed across the training and validation datasets, although the improvement over the best-performing single learner was modest and should be interpreted cautiously (**Table 3**; **Figure 5**).

**Table 3:** Predictive performance of candidate models for comorbid anxiety disorder.

Model	Model structure	AUC (95% CI)	Accuracy (95% CI)	Sensitivity (95% CI)	Specificity (95% CI)	PPV (95% CI)	NPV (95% CI)	F1 score (95% CI)	Brier score (95% CI)
EBM	Explainable boosting machine	0.789 (0.722–0.848)	0.725 (0.657–0.784)	0.667 (0.544–0.771)	0.754 (0.672–0.821)	0.575 (0.461–0.682)	0.819 (0.739–0.878)	0.618 (0.516–0.708)	0.172 (0.151–0.195)
SVM	Calibrated support vector machine	0.775 (0.707–0.836)	0.714 (0.646–0.774)	0.619 (0.496–0.729)	0.762 (0.680–0.828)	0.565 (0.448–0.676)	0.800 (0.720–0.862)	0.591 (0.485–0.684)	0.180 (0.158–0.203)
Random Forest	Regularized bagging ensemble	0.801 (0.737–0.858)	0.741 (0.674–0.798)	0.698 (0.576–0.798)	0.762 (0.680–0.828)	0.595 (0.481–0.699)	0.835 (0.756–0.892)	0.642 (0.542–0.730)	0.169 (0.148–0.192)
CatBoost	Regularized boosting ensemble	0.824 (0.763–0.877)	0.767 (0.702–0.822)	0.746 (0.627–0.837)	0.778 (0.698–0.842)	0.627 (0.514–0.727)	0.860 (0.784–0.912)	0.681 (0.585–0.764)	0.158 (0.137–0.181)
Stacking model	EBM + SVM + RF + CatBoost → L2 Logistic	0.846 (0.789–0.894)	0.788 (0.725–0.841)	0.762 (0.644–0.850)	0.802 (0.723–0.862)	0.658 (0.543–0.756)	0.871 (0.798–0.920)	0.706 (0.611–0.788)	0.146 (0.126–0.169)

Validation-set estimates with 95% CIs; stacking used EBM, SVM, Random Forest, CatBoost and an L2-penalized logistic meta-learner. Classification metrics were calculated using model-specific thresholds determined exclusively within the training cohort. The validation cohort was used only for final performance estimation.

**Figure 5:**
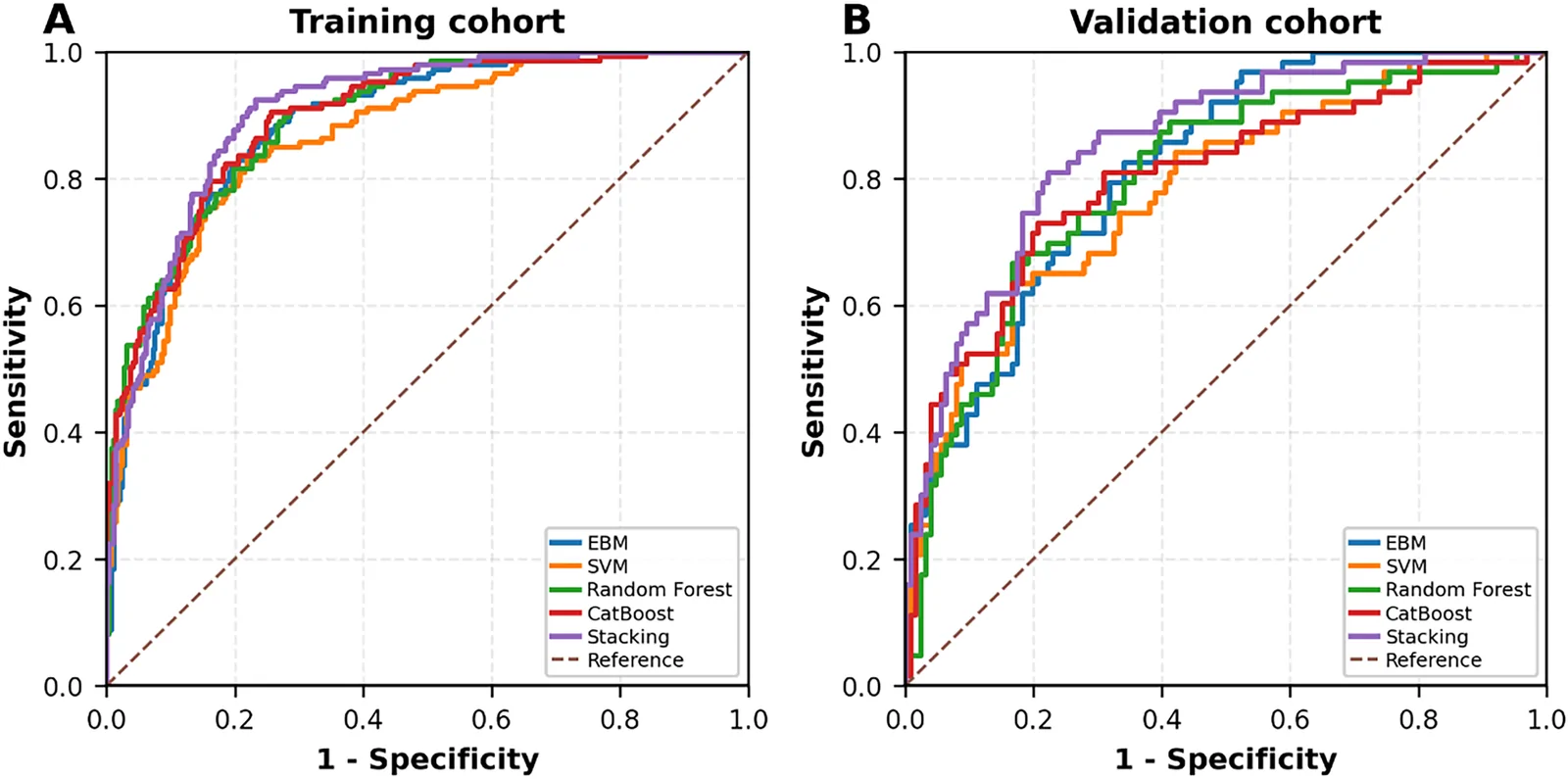
Receiver operating characteristic (ROC) curves comparing the discriminative performance of the base learners and the stacking model in **(A)** the training cohort and **(B)** the validation cohort. The diagonal dashed line represents the reference line.

Decision curve analysis was used to examine the potential clinical utility of the stacking model across different threshold probabilities. Compared with the treat-all and treat-none strategies, the stacking model showed a higher net benefit across a range of clinically relevant thresholds, suggesting that model-guided risk stratification may provide additional value for identifying patients at elevated risk of comorbid anxiety disorder (**Figure 6**). Calibration analysis showed an overall agreement between predicted and observed risks, although mild fluctuation was observed in the validation calibration curve. Consistently, the stacking model had the lowest Brier score among all candidate models in the validation cohort, with a value of 0.146, 95% CI 0.126 to 0.169, supporting its overall probability estimation performance in this internal validation dataset (**Table 3**; **Figure 7**). Pairwise DeLong tests were performed to compare the validation-set AUC of the stacking model with those of the individual base learners (**Table 4**). The stacking model showed significantly higher AUC than EBM (ΔAUC = 0.057, Z = 2.34, P = 0.019) and SVM (ΔAUC = 0.071, Z = 2.79, P = 0.005). The difference between the stacking model and Random Forest was borderline significant (ΔAUC = 0.045, Z = 1.96, P = 0.050). However, compared with CatBoost, the best-performing single learner, the stacking model showed only a modest and non-significant AUC improvement (ΔAUC = 0.022, Z = 1.21, P = 0.226). These findings indicate that although the stacking model achieved the highest numerical AUC, its incremental discrimination advantage over CatBoost was limited and should be interpreted cautiously.

**Figure 6:**
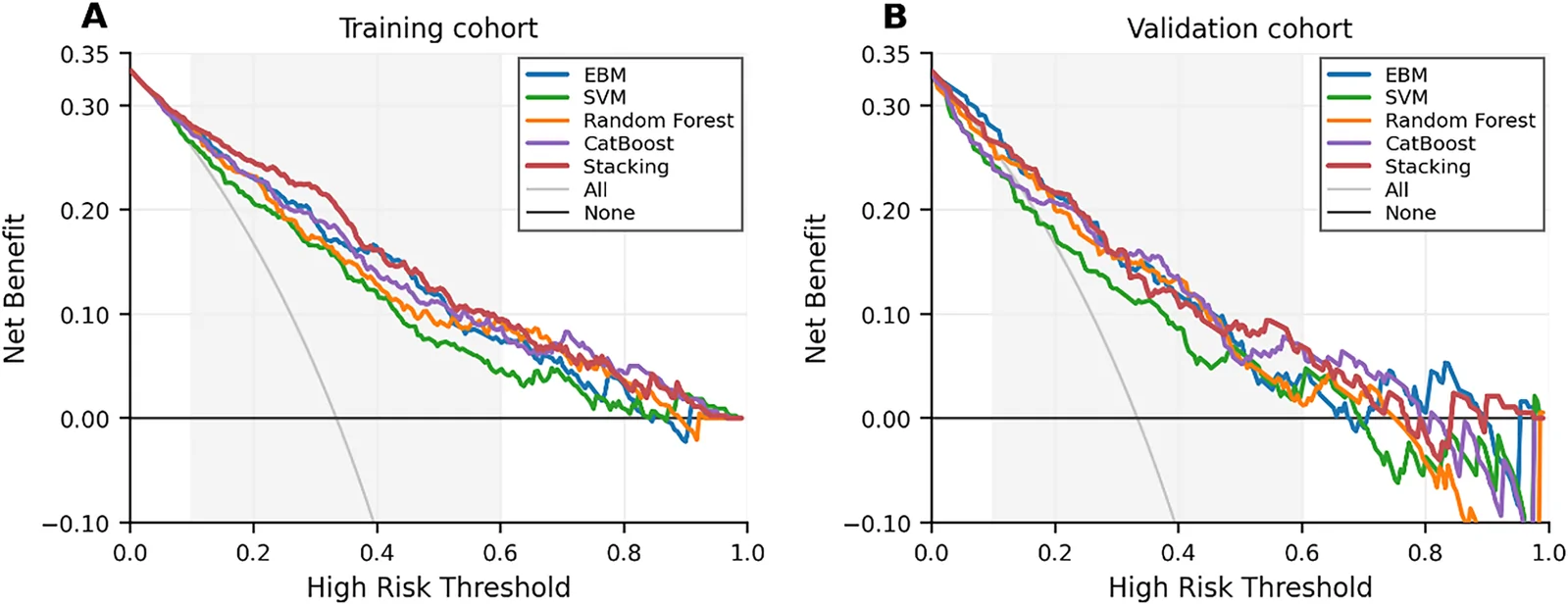
Decision curve analysis comparing the net clinical benefit of the candidate machine learning models across threshold probabilities in **(A)** the training cohort and **(B)** the validation cohort. The treat-all and treat-none strategies are included as references.

**Figure 7:**
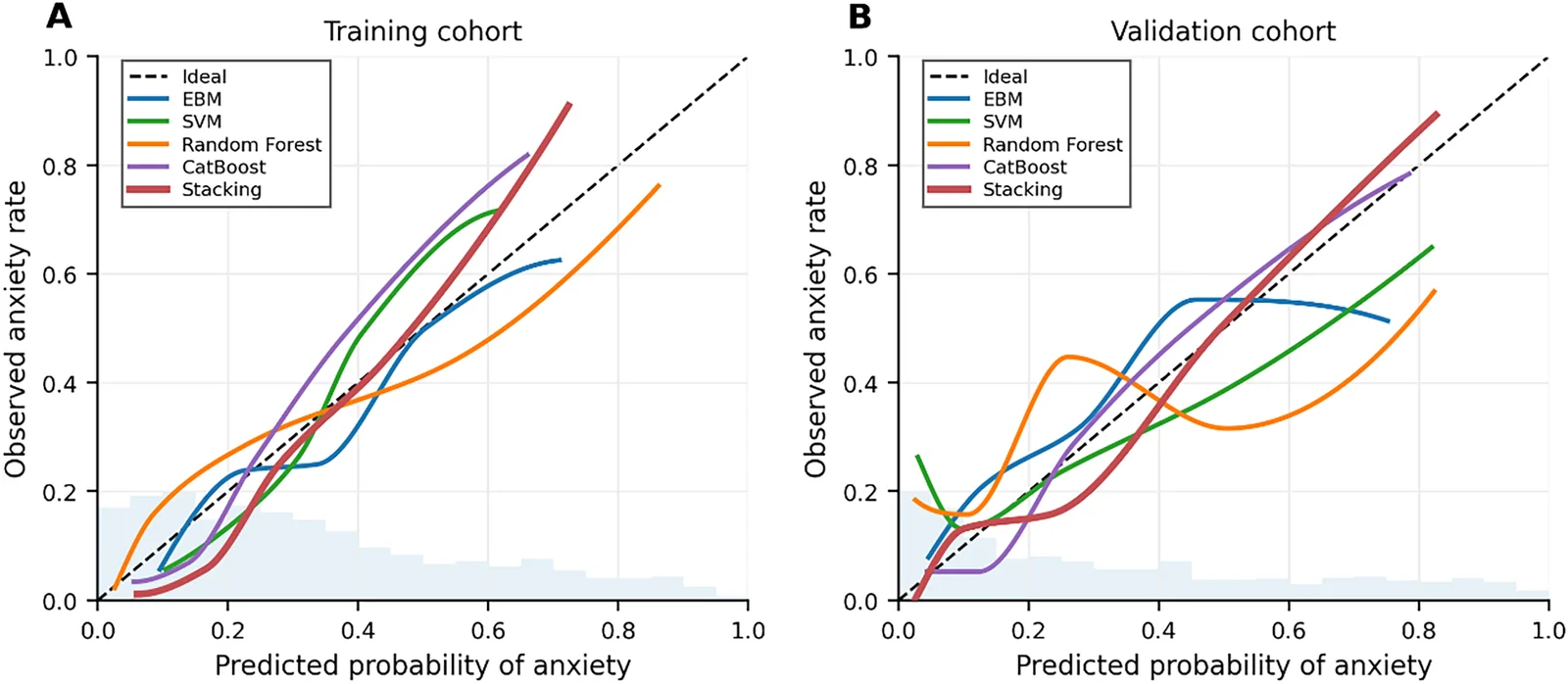
Calibration curves comparing predicted and observed probabilities of comorbid anxiety disorder in **(A)** the training cohort and **(B)** the validation cohort. The diagonal dashed line indicates ideal calibration, with curves closer to this line indicating better agreement between predicted and observed risks.

**Table 4:** Pairwise DeLong test comparisons of validation-set AUCs between the stacking model and individual base learners.

Comparison	AUC of stacking model	AUC of comparator model	ΔAUC	Z statistic	P value
Stacking vs EBM	0.846	0.789	0.057	2.34	0.019
Stacking vs SVM	0.846	0.775	0.071	2.79	0.005
Stacking vs Random Forest	0.846	0.801	0.045	1.96	0.050
Stacking vs CatBoost	0.846	0.824	0.022	1.21	0.226

ΔAUC was calculated as the AUC of the stacking model minus that of the comparator model. Pairwise comparisons of correlated ROC curves were performed using the DeLong test in the validation cohort. All P values were two-sided. EBM, explainable boosting machine; SVM, support vector machine; AUC, area under the receiver operating characteristic curve.

### 3.5 Model interpretability and predictor correlation structure

Model interpretability was assessed using SHAP analysis applied to the complete stacking pipeline, with the original clinical predictors used as explanatory variables. Because the SHAP values were expressed on the log-odds scale of the final stacking model output, positive SHAP values indicate shifts toward a higher estimated probability of comorbid anxiety disorder, whereas negative SHAP values indicate shifts toward a lower estimated probability.

The SHAP summary and feature-importance plots showed that school absence, parental GAD-7 score, CSI-24 score, pain-stress association, symptom duration, pain frequency, SDSC score, age, and night-time awakening contributed most prominently to the final model output. Among these variables, school absence showed the highest mean absolute SHAP value, followed by parental GAD-7 score and CSI-24 score, indicating that school impairment, parental anxiety symptoms, and child somatic symptom burden were the leading contributors to model output. The distribution of SHAP values suggested that higher levels of school absence, parental GAD-7 score, CSI-24 score, pain frequency, symptom duration, and SDSC score generally shifted the model output toward a higher estimated probability of anxiety. The waterfall plot for the patient with the highest predicted risk further illustrated that elevated CSI-24 score, school absence, pain frequency, and pain-stress association were major positive contributors on the log-odds scale, while the force plot demonstrated how individual predictors exerted opposing effects on patient-level model output (**Figure 8**).

**Figure 8:**
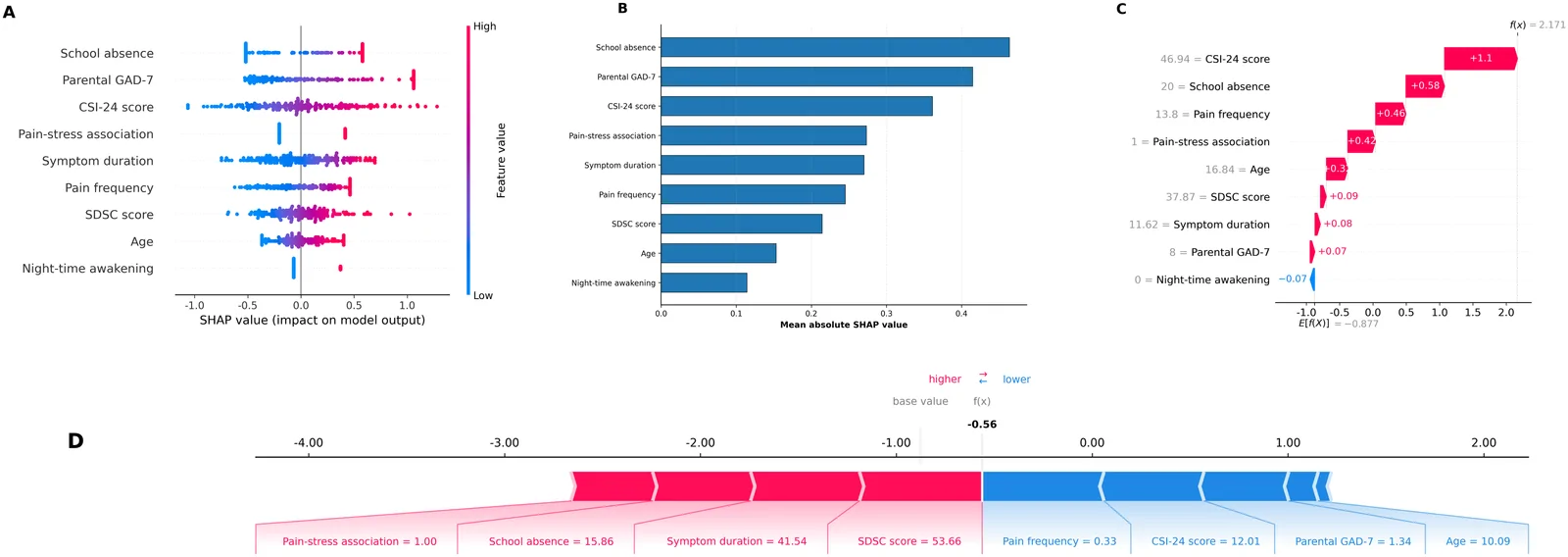
SHAP-based interpretation of the anxiety identification model. **(A)** SHAP summary plot of predictor contributions. **(B)** Global feature importance ranked by mean absolute SHAP value. **(C)** Waterfall plot showing predictor contributions in a high-risk patient. **(D)** Force plot showing predictors that increased or decreased the predicted log-odds of comorbid anxiety disorder. SHAP values are presented on the log-odds scale.

The correlation matrix and pairwise scatterplots were used to examine the dependence structure among selected predictors. Overall, most pairwise Pearson correlation coefficients were weak to moderate, and no correlation exceeded 0.60, indicating no evident severe collinearity among the selected variables. The strongest correlations were observed between school absence and CSI 24 score, r = 0.55, school absence and pain frequency, r = 0.50, and CSI 24 score and pain frequency, r = 0.47. A moderate correlation was also observed between pain stress association and parental GAD 7 score, r = 0.42. Other associations were weaker, including SDSC score with night time awakening, r = 0.29, SDSC score with age, r = 0.28, and CSI 24 score with pain stress association, r = 0.24. Scatterplots colored by predicted anxiety probability showed that higher predicted risks tended to cluster among patients with greater symptom burden, school absence, and family psychological risk indicators, supporting the interpretability patterns observed in the SHAP analysis (**Figure 9**).

**Figure 9:**
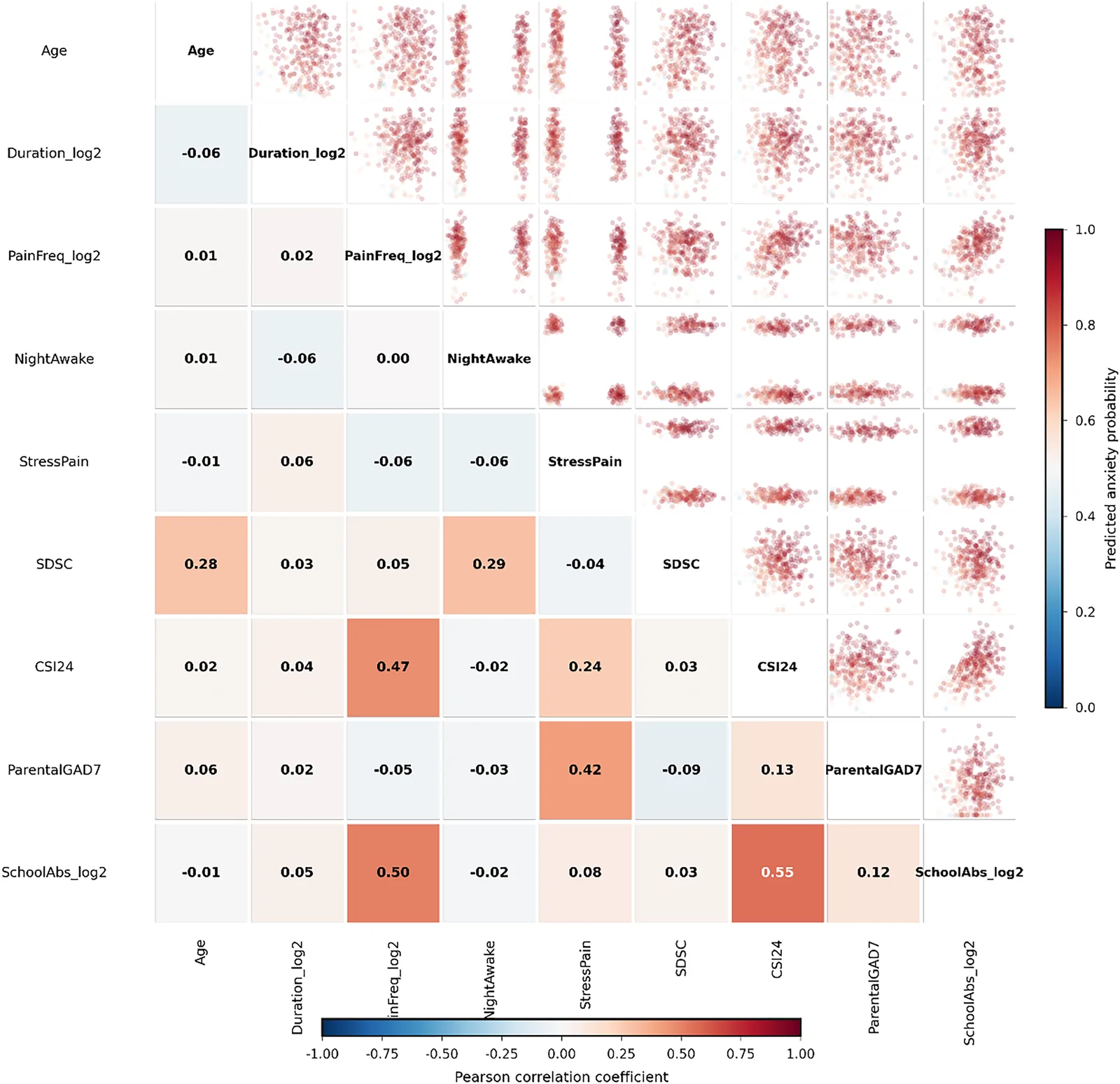
Correlation matrix and pairwise scatterplots of selected predictors, colored by predicted anxiety probability. Lower panels show Pearson correlation coefficients.

## 4 Discussion

In this study, a model incorporating abdominal pain characteristics, child psychosocial and school-related measures, and parent and family factors showed acceptable performance in an internal held-out validation cohort. Although the validation AUC was slightly higher than the out-of-fold training estimate, this pattern should not be taken as evidence of robust generalizability; it may instead reflect sampling variability arising from the modest size of the validation cohort and the use of a single random split. The proposed model should therefore be considered a feasible framework for risk stratification, pending validation in independent cohorts. The final stacking model achieved the highest numerical performance among the evaluated learners, with acceptable calibration and potential clinical utility. The interpretability analyses suggested a clinically plausible risk profile, supporting the view that anxiety in pediatric functional abdominal pain disorders is embedded within a multidimensional biopsychosocial context.

The predictors included in the model reflect clinically relevant domains of biopsychosocial functioning in children and adolescents with functional abdominal pain disorders. Age may capture developmental differences in symptom perception, emotional expression, coping strategies, and the ability to report anxiety-related distress (18, 19). Longer symptom duration and higher abdominal pain frequency indicate greater cumulative pain exposure and symptom burden, which may increase uncertainty, anticipatory worry, and functional limitation (20). Night-time awakening due to pain is clinically important because pain-related sleep disruption may heighten threat appraisal in both children and parents and may further exacerbate anxiety through fatigue, autonomic arousal, impaired emotion regulation, and reduced pain tolerance (21, 22). Abdominal pain temporally associated with stressful events suggests a stress-responsive pain pattern and supports the role of emotional and environmental triggers in symptom exacerbation. The SDSC total score reflects sleep disturbance, whereas the CSI-24 score captures broader somatic symptom burden, potentially indicating somatic amplification, heightened interoceptive attention, or shared vulnerability between anxiety and physical symptoms (23–26). Parental GAD-7 score represents the family emotional context, as parental anxiety may influence symptom interpretation, reassurance seeking, health-care utilization, and avoidance reinforcement (27–29). Finally, school absence provides a pragmatic marker of functional impairment and may reflect avoidance behavior, academic stress, reduced coping capacity, and the broader impact of abdominal pain and anxiety on daily functioning.

Our findings are consistent with recent evidence supporting the use of machine learning for pediatric functional gastrointestinal risk assessment (30). In contrast, our study focused on children and adolescents with functional abdominal pain disorders and aimed to identify comorbid anxiety disorder rather than the onset of gastrointestinal symptoms. By integrating pain characteristics, sleep disturbance, somatic symptoms, school impairment, and parental anxiety within a stacking ensemble framework, our model emphasizes the biopsychosocial nature of anxiety risk in older pediatric patients with disorders of gut-brain interaction.

The study also incorporated several safeguards relevant to clinical machine learning. The model was developed from clinically available variables measured before outcome classification, while anxiety-specific diagnostic scores and post-diagnostic variables were excluded to limit information leakage. The held-out validation cohort was not used for feature selection, hyperparameter tuning, calibration, threshold selection, base-learner specification, or meta-learner training. In addition, stacking was based on out-of-fold predicted probabilities, reducing the risk that the meta-learner learned from overly optimistic in-sample predictions. The use of heterogeneous base learners and formal diversity analyses further supported the rationale for an ensemble approach. Importantly, evaluation extended beyond discrimination to include calibration, Brier score, threshold-dependent performance, decision curve analysis, and SHAP-based interpretation, which are central considerations for clinical models intended to support risk stratification.

This study has several limitations. First, the model was developed and evaluated using internal validation only; external validation across independent centers, regions, and care settings is needed to assess transportability before clinical implementation. Second, the observational design precludes causal interpretation. Variables such as school absence, parental anxiety, sleep disturbance, and somatic symptom burden should be viewed as correlates of estimated anxiety probability rather than causal risk factors. Third, selection bias is possible because the analysis included only participants who met eligibility criteria and had sufficient clinical, psychosocial, and family-level data. Fourth, despite efforts to reduce information leakage, the sample size was moderate for a complex ensemble-learning framework, leaving uncertainty around subgroup performance, calibration, and threshold-dependent estimates. Fifth, anxiety assessment may vary across clinicians and settings; future studies should further standardize diagnostic procedures and assess inter-rater reliability when feasible. Finally, SHAP analysis explains model behavior but does not establish causal mechanisms, and this study did not evaluate whether model-guided risk stratification improves referral decisions, treatment uptake, clinical outcomes, school functioning, or cost-effectiveness. Prospective impact studies are therefore required before this approach can be incorporated into routine clinical pathways.

## 5 Conclusion

In this study, a stacking machine learning model incorporating pain characteristics, child psychosocial and school-related variables, and family factors showed good performance in identifying comorbid anxiety disorder in children and adolescents with functional abdominal pain disorders. These findings suggest that anxiety risk reflects a multidimensional biopsychosocial profile involving school impairment, somatic symptoms, sleep disturbance, stress-related pain, and parental anxiety. The model may facilitate early risk stratification and guide referral for psychological assessment, although external validation and prospective impact studies are required before clinical implementation.

## Data Availability

The original contributions presented in the study are included in the article/**Supplementary Material**. Further inquiries can be directed to the corresponding author.
