# Supplementary material for: A stacking machine learning model for identifying comorbid anxiety disorder in children and adolescents with functional abdominal pain disorders
Source: Front Psychiatry. 2026 Oct 23;17:1904174. doi: 10.3389/fpsyt.2026.1904174 (PMC13739682; doi:10.3389/fpsyt.2026.1904174)
Supplement: Supplementary file 1 [file Supplementaryfile1.docx]

**Table S1. Missingness and imputation of candidate predictors and the outcome in the final analytic cohort.**

| **Candidate variable** | **Missing, n (%)** | **Imputation method** |
| --- | --- | --- |
| Age, years | 0 (0.0%) | No imputation |
| Sex | 0 (0.0%) | No imputation |
| Body mass index, kg/m² | 5 (0.8%) | Training-derived median |
| Urban residence | 2 (0.3%) | Training-derived mode |
| Only-child status | 3 (0.5%) | Training-derived mode |
| Symptom duration, months | 4 (0.6%) | Training-derived median |
| Abdominal pain frequency, episodes/week | 6 (1.0%) | Training-derived median |
| Abdominal pain intensity, NRS score | 5 (0.8%) | Training-derived median |
| Morning abdominal pain | 2 (0.3%) | Training-derived mode |
| Night-time awakening due to pain | 1 (0.2%) | Training-derived mode |
| Abdominal pain temporally associated with stressful events | 7 (1.1%) | Training-derived mode |
| Functional Disability Inventory score | 10 (1.6%) | Training-derived median |
| School absence, days/month | 8 (1.3%) | Training-derived median |
| Previous emergency visit for abdominal pain | 0 (0.0%) | No imputation |
| Previous gastrointestinal medication use | 1 (0.2%) | Training-derived mode |
| Functional abdominal pain disorder subtype | 0 (0.0%) | No imputation |
| Constipation | 1 (0.2%) | Training-derived mode |
| Diarrhoea | 1 (0.2%) | Training-derived mode |
| Nausea | 1 (0.2%) | Training-derived mode |
| Abdominal bloating | 2 (0.3%) | Training-derived mode |
| Headache or migraine | 2 (0.3%) | Training-derived mode |
| Allergic diseases | 3 (0.5%) | Training-derived mode |
| SDSC total score | 9 (1.4%) | Training-derived median |
| School avoidance | 7 (1.1%) | Training-derived mode |
| Peer relationship difficulties | 8 (1.3%) | Training-derived mode |
| Recent academic stress | 10 (1.6%) | Training-derived mode |
| CSI-24 score | 11 (1.7%) | Training-derived median |
| Child pain catastrophizing score | 12 (1.9%) | Training-derived median |
| Child depressive symptom score | 13 (2.1%) | Training-derived median |
| Primary caregiver | 1 (0.2%) | Training-derived mode |
| Single-parent family | 0 (0.0%) | No imputation |
| Left-behind child status | 2 (0.3%) | Training-derived mode |
| Maternal education | 6 (1.0%) | Training-derived mode |
| Paternal education | 8 (1.3%) | Training-derived mode |
| Monthly household income relative to local median | 20 (3.2%) | Training-derived mode |
| Parental marital conflict | 9 (1.4%) | Training-derived mode |
| Parental GAD-7 score | 12 (1.9%) | Training-derived median |
| Parental depressive symptoms | 11 (1.7%) | Training-derived mode |
| Parental history of anxiety or depression | 6 (1.0%) | Training-derived mode |
| Family history of psychiatric disorders | 5 (0.8%) | Training-derived mode |
| Parental pain catastrophizing score | 14 (2.2%) | Training-derived median |
| Overprotective parenting style | 9 (1.4%) | Training-derived mode |
| High parental health-care seeking tendency | 7 (1.1%) | Training-derived mode |
| Family history of recurrent abdominal pain | 4 (0.6%) | Training-derived mode |

**Note:** Continuous variables with residual missingness were imputed using the median estimated from the corresponding training data, whereas categorical variables were imputed using the most frequent category. During cross-validation, imputation parameters were estimated separately within each training fold and then applied to the corresponding held-out fold. For final validation-set prediction, imputation parameters estimated from the full training cohort were applied unchanged to the validation cohort. The outcome variable had no missing values and was not imputed.

**Table S2. Out-of-fold cross-validation performance in the training cohort**

| **Model** | **Model structure** | **AUC (95% CI)** | **Accuracy (95% CI)** | **Sensitivity (95% CI)** | **Specificity (95% CI)** | **Brier score (95% CI)** |
| --- | --- | --- | --- | --- | --- | --- |
| EBM | Explainable boosting machine | 0.776 (0.728–0.820) | 0.716 (0.672–0.758) | 0.667 (0.587–0.740) | 0.740 (0.685–0.790) | 0.176 (0.160–0.194) |
| SVM | Standardized and calibrated support vector machine | 0.762 (0.713–0.807) | 0.704 (0.659–0.746) | 0.653 (0.572–0.727) | 0.730 (0.675–0.780) | 0.185 (0.168–0.203) |
| Random Forest | Regularized bagging tree ensemble | 0.786 (0.739–0.828) | 0.725 (0.681–0.766) | 0.680 (0.600–0.751) | 0.747 (0.693–0.796) | 0.174 (0.157–0.192) |
| CatBoost | Regularized gradient boosting model | 0.808 (0.763–0.848) | 0.748 (0.705–0.787) | 0.707 (0.628–0.775) | 0.768 (0.716–0.815) | 0.163 (0.147–0.181) |
| Stacking model | EBM + SVM + RF + CatBoost → L2-penalized logistic regression | 0.831 (0.789–0.868) | 0.768 (0.726–0.806) | 0.735 (0.658–0.800) | 0.785 (0.734–0.830) | 0.151 (0.135–0.169) |

**Note:** OOF estimates were generated only within the training cohort. In each cross-validation split, all preprocessing, feature selection, tuning, calibration, and threshold selection were performed using the analysis fold only; the held-out fold was used solely for OOF prediction. The validation cohort was not used for model development, tuning, calibration, threshold selection, or meta-learner training.

**Table S3. Penalized meta-learner coefficients and standardized contributions of base learners in the stacking model**

| Meta-feature | Penalized β coefficient | exp(β) | SD of OOF predicted probability | Standardized contribution, β × SD | Relative contribution, % | Relative contribution ranking |
| --- | --- | --- | --- | --- | --- | --- |
| EBM predicted probability | 0.55 | 1.73 | 0.19 | 0.105 | 25.8 | 2 |
| SVM predicted probability | 0.42 | 1.52 | 0.17 | 0.071 | 17.7 | 4 |
| Random Forest predicted probability | 0.51 | 1.67 | 0.18 | 0.092 | 22.7 | 3 |
| CatBoost predicted probability | 0.76 | 2.14 | 0.18 | 0.137 | 33.8 | 1 |

**Note:** EBM, explainable boosting machine; SVM, support vector machine; OOF, out-of-fold. The L2-penalized logistic meta-learner used OOF base-learner predicted probabilities as meta-features. Penalized coefficients, exp(β), standardized contribution, and relative contribution are presented descriptively to indicate each base learner’s direction and relative contribution to the stacked prediction. Exp(β) values should not be interpreted as clinical odds ratios or inferential effect estimates. Because the meta-learner was penalized and meta-features were correlated, Wald-type 95% confidence intervals and P values were not reported.

**Table S4. Pairwise diversity metrics among base learners**

| Model pair | Correlation of OOF probabilities | Disagreement | Cohen’s kappa | Double-fault | Yule’s Q |
| --- | --- | --- | --- | --- | --- |
| EBM vs SVM | 0.62 | 0.28 | 0.42 | 0.150 | 0.47 |
| EBM vs Random Forest | 0.58 | 0.31 | 0.37 | 0.125 | 0.41 |
| EBM vs CatBoost | 0.55 | 0.33 | 0.34 | 0.103 | 0.38 |
| SVM vs Random Forest | 0.60 | 0.29 | 0.40 | 0.141 | 0.44 |
| SVM vs CatBoost | 0.57 | 0.32 | 0.36 | 0.114 | 0.39 |
| Random Forest vs CatBoost | 0.68 | 0.24 | 0.51 | 0.144 | 0.56 |

**Note:** OOF, out-of-fold; EBM, explainable boosting machine; SVM, support vector machine. Diversity metrics were calculated from OOF probabilities or paired binary predictions to assess whether base learners produced non-identical and complementary outputs before stacking.

**Table S5. Out-of-fold learner-diversity and performance measures of candidate stacking combinations in the training cohort**

| Base learners | Mean pairwise probability correlation | Mean pairwise disagreement | Mean pairwise double-fault rate | Mean entropy-based diversity | OOF AUC | OOF F1 score | OOF Brier score |
| --- | --- | --- | --- | --- | --- | --- | --- |
| EBM + SVM | 0.62 | 0.28 | 0.150 | 0.63 | 0.812 | 0.651 | 0.161 |
| RF + CatBoost | 0.68 | 0.24 | 0.144 | 0.58 | 0.835 | 0.676 | 0.153 |
| EBM + RF + CatBoost | 0.60 | 0.29 | 0.124 | 0.66 | 0.840 | 0.683 | 0.151 |
| EBM + SVM + CatBoost | 0.58 | 0.31 | 0.122 | 0.69 | 0.842 | 0.688 | 0.149 |
| EBM + SVM + RF + CatBoost | 0.60 | 0.30 | 0.130 | 0.72 | 0.831 | 0.679 | 0.151 |

**Note:** All measures were calculated exclusively from out-of-fold predictions in the training cohort. For combinations containing three or more learners, pairwise diversity measures were calculated for all possible learner pairs and summarized as the mean pairwise value. Lower probability correlation, lower double-fault rate, and lower Brier score indicate more favorable values, whereas higher disagreement, entropy-based diversity, AUC, and F1 score indicate more favorable values. These analyses were descriptive and were not used for final model selection.

**Figure S1.** Pairwise diversity among base learners was assessed using OOF probability correlation, prediction disagreement, and double-fault rate.
